# Bone loss and aggravated autoimmune arthritis in HLA-DRβ1-bearing humanized mice following oral challenge with *Porphyromonas gingivalis*

**DOI:** 10.1186/s13075-016-1143-6

**Published:** 2016-10-26

**Authors:** Indra Sandal, Anastasios Karydis, Jiwen Luo, Amanda Prislovsky, Karen B. Whittington, Edward F. Rosloniec, Chen Dong, Deborah V. Novack, Piotr Mydel, Song Guo Zheng, Marko Z. Radic, David D. Brand

**Affiliations:** 1 Research Service, Memphis VA Medical Center, 1030 Jefferson Avenue, Memphis, TN 38104 USA; 2 Department of Periodontology, University of Tennessee Health Science Center, Memphis, TN 38163 USA; 3 Department of Medicine, University of Tennessee Health Science Center, Memphis, TN 38163 USA; 4 Department of Pathology, University of Tennessee Health Science Center, Memphis, TN 38163 USA; 5 Institute for Immunology, Tsinghua University, Beijing, 100084 China; 6 Musculoskeletal Research Center, Departments of Medicine and Pathology, Washington University School of Medicine, St. Louis, MO 63110 USA; 7 Broegelmann Research Laboratory, Department of Clinical Science, University of Bergen, Bergen, Norway; 8 Division of Rheumatology, Department of Medicine, Pennsylvania State University Hershey College of Medicine, Hershey, PA 17033 USA; 9 Department of Microbiology Immunology and Biochemistry, University of Tennessee Health Science Center, Memphis, TN 38163 USA

**Keywords:** Periodontal disease, Rheumatoid arthritis, *Porphyromonas gingivalis*, Animal model

## Abstract

**Background:**

The linkage between periodontal disease and rheumatoid arthritis is well established. Commonalities among the two are that both are chronic inflammatory diseases characterized by bone loss, an association with the shared epitope susceptibility allele, and anti-citrullinated protein antibodies.

**Methods:**

To explore immune mechanisms that may connect the two seemingly disparate disorders, we measured host immune responses including T-cell phenotype and anti-citrullinated protein antibody production in human leukocyte antigen (HLA)-DR1 humanized C57BL/6 mice following exposure to the Gram-negative anaerobic periodontal disease pathogen *Porphyromonas gingivalis.* We measured autoimmune arthritis disease expression in mice exposed to *P. gingivalis*, and also in arthritis-resistant mice by flow cytometry and multiplex cytokine-linked and enzyme-linked immunosorbent assays. We also measured femoral bone density by microcomputed tomography and systemic cytokine production.

**Results:**

Exposure of the gingiva of DR1 mice to *P. gingivalis* results in a transient increase in the percentage of Th17 cells, both in peripheral blood and cervical lymph nodes, a burst of systemic cytokine activity, a loss in femoral bone density, and the generation of anti-citrullinated protein antibodies. Importantly, these antibodies are not produced in response to *P. gingivalis* treatment of wild-type C57BL/6 mice, and *P. gingivalis* exposure triggered expression of arthritis in arthritis-resistant mice.

**Conclusions:**

Exposure of gingival tissues to *P. gingivalis* has systemic effects that can result in disease pathology in tissues that are spatially removed from the initial site of infection, providing evidence for systemic effects of this periodontal pathogen. The elicitation of anti-citrullinated protein antibodies in an HLA-DR1-restricted fashion by mice exposed to *P. gingivalis* provides support for the role of the shared epitope in both periodontal disease and rheumatoid arthritis. The ability of *P. gingivalis* to induce disease expression in arthritis-resistant mice provides support for the idea that periodontal infection may be able to trigger autoimmunity if other disease-eliciting factors are already present.

## Background

Rheumatoid arthritis (RA) is a chronic, destructive proinflammatory autoimmune disease with an as yet unknown etiology. RA has both a genetic basis and lifestyle components such as smoking. Among the genetic components, expression of specific polymorphisms of human leukocyte antigen (HLA)-DRβ1 (the so-called “shared epitope” (SE)) is most frequently cited as enhancing the risk of RA and predicting a worse outcome in individuals expressing one or more susceptibility alleles that encode a linear sequence of amino acids in the DRβ1 chain of the HLA‐DRα/β heterodimer between amino acids 67 and 74 (QKRAA in *0401, QRRAA in *0404, *0405, and *0101, and RRRAA in *1001) [1].

Periodontitis (PD) is a chronic inflammatory condition in the periodontal tissues (gingiva, ligament, alveolar bone) that shares many features in common with RA. Like RA, PD results from chronic inflammation, in this case directed toward gingival epithelium and spreading to the underlying connective tissue. As in RA, it also results in bone destruction, in this case alveolar bone supporting the teeth. The pathology and inflammation in both RA and PD are thought to be driven by proinflammatory Th17 cells which can be found both in the RA synovial tissues and in inflamed gingiva [2]. While the linkage between RA and PD is well documented (for review, see [3]), studies elucidating the mechanism(s) driving this linkage are lacking.

One of the best studied periodontal pathogens is an anaerobic, Gram-negative prokaryote known as *Porphyromonas gingivalis*. This organism is a biofilm-forming keystone-pathogen member of the so-called “red-complex” of periodontal pathogens [4]. A recent study [5] found that severity of PD and RA is related, in that RA patients with severe PD have a more robust antibody response against *P. gingivalis* than non-RA controls.

Another feature common to both RA and PD is the generation of antibodies directed against citrullinated proteins. Proteins are citrullinated by the enzyme peptidyl arginine deiminase (PAD) which deiminates the side chain of arginine residues, converting them to citrulline. This conversion results in the generation of neoepitopes believed to induce the production of anti-citrullinated protein antibodies (ACPAs). ACPAs are now used widely as a diagnostic marker for RA because they are highly predictive of disease and are a very early marker that can be detected long before the clinical expression of RA [6]. ACPAs can also be detected in the serum of patients with periodontal disease [7]. It is therefore of great interest that *P. gingivalis* is the only known prokaryote that encodes a PAD enzyme in its genome [8], and is known both to autocitrullinate and to modify host proteins as well [9].

We and others have shown that treatment with *P. gingivalis* can alter the course of experimental arthritis [10–13], and that a mouse which expresses human HLA-DRβ1 as a transgene on the C57BL/6 background reliably develops a high incidence of collagen-induced arthritis. The use of HLA-DRβ1 humanized C57BL/6 mice allowed us to ask whether the DRβ1 transgene might also alter the host response to *P. gingivalis*. Our studies demonstrate that brushing the oral cavity of the B6.DR1 gingiva with *P. gingivalis* results in a transient increase in the percentage of Th17 cells in peripheral blood and in cervical lymph nodes, a burst of systemic cytokine activity, and generation of ACPAs. Importantly, ACPAs produced in response to treatment with *P. gingivalis* are generated only by DRβ1-bearing mice and not in C57BL/6 (WT) mice.

We also analyzed how this response impacted the development of an ongoing autoimmune arthritis. We determined that *P. gingivalis* treatment of mice which had been challenged with type II collagen (CII) emulsified in Complete Freund’s Adjuvant (CFA) resulted in a dramatic hastening of disease onset, increased incidence, and enhanced severity of collagen-induced arthritis. Microcomputed tomographic (μCT) analyses of nonarthritic manus from mice brushed with *P. gingivalis* showed a trend towards decreased bone density relative to manus from unbrushed control mice, but once arthritis was triggered both groups demonstrated an enhanced bone loss that resulted in destruction of the form and function of the bones analyzed.

Lastly, we also found that exposure of arthritis-resistant mice (e.g., mice which had resisted the development of disease expression for months after others in the cohort had developed disease) to *P. gingivalis* can serve as a trigger that breaks their resistance and results in the expression of overt clinical autoimmune arthritis.

These findings suggest that in the context of the appropriate susceptibility allele, infection with a red-complex oral pathogen such as *P. gingivalis* may serve as an important factor that can tip the balance in favor of autoimmunity and can either exacerbate existing disease or provide the necessary impetus to drive overt expression of subclinical disease processes.

## Methods

### Animals

We developed an I-A°/I-E° [14] mouse on the C57BL/6 background that expresses a chimeric mouse/human RA/PD susceptibility allele HLA-DRβ1(*0101) as a transgene as described previously [15]. Using a Foxp3^gfp^ reporter (kind gift from Alexander Rudensky [16]) and an IL-17F^mrfp^ reporter developed earlier [17], B6.DR1 mice were crossed to facilitate the flow cytometric identification and isolation of Treg and Th17 cells. Mice were carefully screened to ensure the presence of all transgenes as well as the absence of murine class II. All studies were performed under protocol 316941 which was approved by the Institutional Animal Care and Use Committee at the Memphis VA Medical Center.

### Bacterial culture

*P. gingivalis* strain W83 was grown overnight in ATCC 2722 medium: tryptic soy broth supplemented with hemin (5 mg/ml) and menadione (0.5 mg/ml) at 37 °C in an anaerobic chamber equilibrated with a mixture of 90 % nitrogen, 5 % carbon dioxide, and 5 % hydrogen. Bacterial cell counts were determined using a spectrophotometer with an optical density of 1.0 at 600 nm corresponding to 1 × 10^9^ CFU/ml. Then 10^9^ CFU/ml of bacteria were harvested and washed three times in PBS, and resuspended at 3.33 × 10^7^/ml in PBS with 2 % carboxymethylcellulose (CMC).

### Bacterial inoculation

Briefly, 10-12 week old B6.DR1 mice and/or C57BL/6 wild-type (WT) mice received Bactrim (sulfamethoxizole/trimethoprim) in deionized water for 7 days. Three days after the antibiotic treatment the mice were put under brief isoflurane anesthesia, and 10^6^ CFU of live *P. gingivalis* in 30 μl of PBS with 2 % CMC were administered using a modification of the brush method used by Cantley et al. [10]. Instead of dipping a brush in a solution of *P. gingivalis* in CMC, we delivered the slurry through customized tuberculin syringes to which plastic bristles had been affixed in place of a needle. *P. gingivalis* was applied to the gingival margin of mouse maxillary molars daily for 7 days. A sham infected group received 30 μl of PBS with 2 % CMC alone. In arthritis experiments, mice that had been challenged with CII emulsified in CFA were brushed with 10^6^ CFU of live *P. gingivalis* in 30 μl of PBS with 2 % CMC without prior Bactrim treatment.

### Flow cytometry

At specific time points before, during, and after brushing with live *P. gingivalis*, peripheral blood was drawn and stained with the following antibodies: Alexafluor 700 conjugated anti-mouse CD3ε, Peridinin Clorophyll/Cyanine 5.5 conjugated anti-mouse CD45, Pacific Blue conjugated anti-mouse CD4, and Phycoerythrin/Cyanine 7 conjugated anti-mouse CD25 (all BD Biosciences) prior to acquisition on a SORP 5-Laser LSR II (BD Immunocytometry Systems). In some experiments, cervical lymph nodes were obtained at the time of sacrifice and were subjected to the same panel of antibodies. Analysis was performed using FlowJo v10.1.

### μCT analysis

Legs were harvested and fixed in 10 % neutral buffered formalin solution for 72 hours and transferred to PBS prior to dissecting femurs from the other leg tissues. They were then stored and imaged in 70 % EtOH and scanned with the following settings: 60 kV, 167 μA, 0.5 mm aluminum filter, 0.7° rotation step, 4 frames averaging 2000 × 1336 CCD, 800 msec exposure, and 10 μm voxel size on a Skyscan 1172 instrument. Scan time was 34 minutes.

The distal end of the volume of interest (VOI) started 1.5 mm proximally from the distal growth plate. It continued proximally for 1.5 mm. Trabecular bone was isolated using an automated script and 3D morphometric analyses were performed.

For evaluation of cortical bone, the midpoint of the VOI was located at 55 % of the total length of the femur from the proximal end. The total length of the VOI was 1 mm. Cortical bone was isolated using an automated script. 3D and 2D morphometric analyses were performed. The 3D and 2D VOIs correspond to those described by Bouxsein et al. [18].

Paw and whole leg scans were performed on a Scanco μCT 40 using legs fixed in 10 % neutral buffered formalin. They were imaged at an 8 μm voxel size at 55 kV with 145 μA over about 45 minutes.

### Detection of antibodies to citrullinated protein antigens

WT (C57BL/6) mice or B6.DR1 mice (*n* = 5/group) were brushed with live cultures of either WT *P. gingivalis* or a PAD^null^ mutant. Serum samples were obtained at the times indicated and subjected to a commercial (Axis-Shield, Alere, UK) ELISA according to the manufacturer’s instructions, including the selection of the appropriate mouse secondary detection reagent.

### Detection of *P. gingivalis* in blood and maxilla

Blood samples were obtained from B6.DR1 mice brushed with live cultures of *P. gingivalis* or those left untreated. Maxillae were harvested from mice 3 months following the last treatment and were crushed under liquid nitrogen in a mortar and pestle. Genomic DNA was obtained using a DNeasy Blood & Tissue Kit according to the manufacturer’s instructions (Qiagen). PCR was performed according to the methods of Marchesan et al. [19].

### Cytokine analysis

Cytokines were measured in serum samples using the Mouse Th17 6-plex (Bio-Rad) according to the manufacturer’s instructions and using internal standards supplied with the kit. The limits of detection (in pg/ml) for each analyte were as follows: IL-1β, 9.4; IL-6, 0.2; IL-10, 1.0; IL-17A, 0.8; IFN-γ, 1.2; and TNF-α, 1.4.

### Collagen-induced arthritis

Arthritis was induced in B6.DR1 mice following our well-established protocol [20]. Thirty-nine B6.DR1 mice were challenged with 100 μg of native bovine type II collagen (extracted and purified in our laboratory) emulsified in CFA made from 15 % (v/v) mannide monooleate in heavy mineral oil. When the first clinical signs of disease expression (paw swelling) were observed at day 26, cultures of *P. gingivalis* were initiated and the first *P. gingivalis* treatments were started on 20 of the mice. Treatments were performed daily for 7 days. Mice were visually assessed for arthritis [20] both during and after brushing.

## Results

### Rise in Th17 cell expression following treatment with *P. gingivalis*

**Fig. 1:**
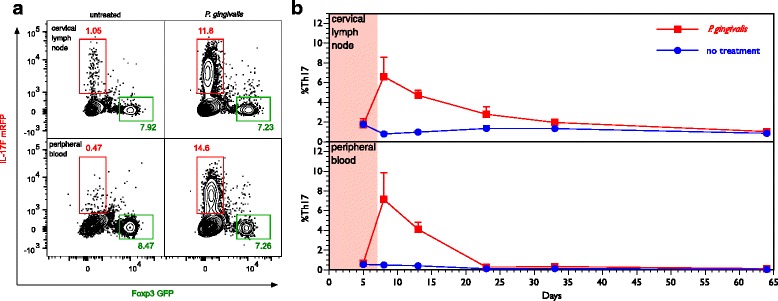
Th17 response to oral challenge with live *P. gingivalis.* Healthy suspensions of live *P. gingivalis* were mixed with CMC and brushed onto the gingival tissues of B6.DR1 mice (*shaded pink area*). **a** Contour plots for flow cytometric analysis of cervical lymph nodes and peripheral blood mononuclear cells from individual B6.DR1 mice with or without treatment with *P. gingivalis*. Plots are gated on CD3^+^CD4^+^ T cells. *Red* numbers in each quadrant represent the percentage of CD3^+^CD4^+^ T cells expressing the IL-17F^mrfp^ reporter, *green* numbers represent the percentage of CD3^+^CD4^+^ T cells expressing the Foxp3^gfp^ reporter. **b** Compilation of Th17 data over the course of 2 months after treatment. Results are expressed as mean percentage of CD3^+^CD4^+^ T cells expressing IL-17F^mrfp^. *N* = 3 or 4 mice at each time point. *Error bars* = SEM. *GFP* green fluorescent protein, *mRFP* mono-red fluorescent protein (Color figure online)

Flow cytometric analyses revealed that brushing the gingival tissues of B6.DR1 mice resulted in a transient increase in the percentage of circulating Th17 cells among the peripheral blood mononuclear cells (PBMCs) relative to untreated mice (Fig. 1). At baseline, less than 1 % of the CD4^+^ T cells in the PBMCs were Th17-positive whereas the level transiently rises to approximately 7 % by the first day after the last of seven daily brushings before falling back to baseline. These findings were similar in the cervical lymph nodes draining the oral cavity, although the return to baseline proceeded more gradually. Using a PCR-based detection method [19], we found evidence of *P. gingivalis* in the blood of B6.DR1 mice at 2 weeks and in the maxilla 3 months after the last inoculation (data not shown). There was no apparent change in the percentage of regulatory T cells expressing Foxp3.

### Production of ACPAs in response to treatment with *P. gingivalis*

**Fig. 2:**
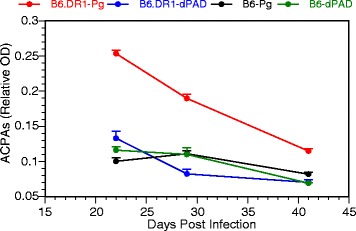
Generation of antibodies to citrullinated protein antigens is enhanced in mice bearing the transgene for human HLA-DR1β, and requires PAD enzyme*.* Healthy suspensions of live *P. gingivalis* or a PAD^null^ mutant strain (*dPAD*) were mixed with CMC and brushed onto the gingival tissues of dual-reporter B6.DR1 mice or WT C57BL/6 mice. Serum samples were obtained 3, 4, and 6 weeks after brushing and applied to commercial ACPA ELISA according to the manufacturer’s instructions. Unbrushed mice generated no detectable ACPAs. The supplied human secondary detection reagent was replaced with an anti-mouse IgG_2b_ antibody detection reagent suggested by the manufacturer. *N* = 5 for each group. *Error bars* = SEM. *ACPA* anti-citrullinated protein antibody, *OD* optical density

We measured ACPAs in sera from B6.DR1 mice treated either with WT (W83) *P. gingivalis* or a PAD^null^ mutant (dPAD) using CCP2 ELISA (Axis-Shield, Alere, UK). The sera were paired with those of WT C57BL/6 (B6) mice brushed with either form of *P. gingivalis* to determine the influence of the HLA-DRβ1 restriction element on the production of ACPAs. We found (Fig. 2) that, relative to B6 mice, B6.DR1 mice produced detectable levels of ACPAs at 3, 4, and 8 weeks after brushing, but only when brushed with a PAD^+^ form of *P. gingivalis*. Our data indicated a direct relationship between HLA genotype and the production of ACPA as well as demonstrating a role for prokaryotic PAD enzyme in this process.

### Cytokine production in response to treatment with *P. gingivalis*

**Fig. 3:**
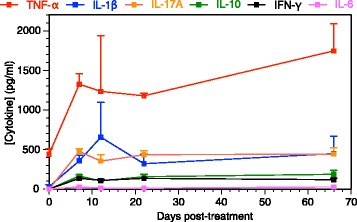
Systemic proinflammatory cytokine response in *P. gingivalis*-brushed mice. Serum samples from B6.DR1 mice brushed daily with *P. gingivalis* were subjected to multiplex cytokine analyses. Cytokines measured at day 0 represent those found in B6.DR1 mice prior to *P. gingivalis* exposure. Limit of detection for each analyte was between 0.8 and 10 pg/ml. *Error bars* = SEM

Serum samples from B6.DR1 mice were also used to measure TNF-α, IL-1β, IL-17A, IL-10, IFN-γ, and IL-6 by multiplex cytokine analysis. We chose serial time points in the weeks after the 7 days of treatment, but also one at 9 weeks after the last treatment. We found (Fig. 3) that relative to baseline values of the animals before treatment (day 0), three of the proinflammatory cytokines TNF-α, IL-1β, and IL-17A were elevated in *P. gingivalis*-treated mice, whereas IL-10, IFN-γ, and IL-6 showed subtle or no changes.

### Bone loss in B6.DR1 mice following treatment with *P. gingivalis*

**Fig. 4:**
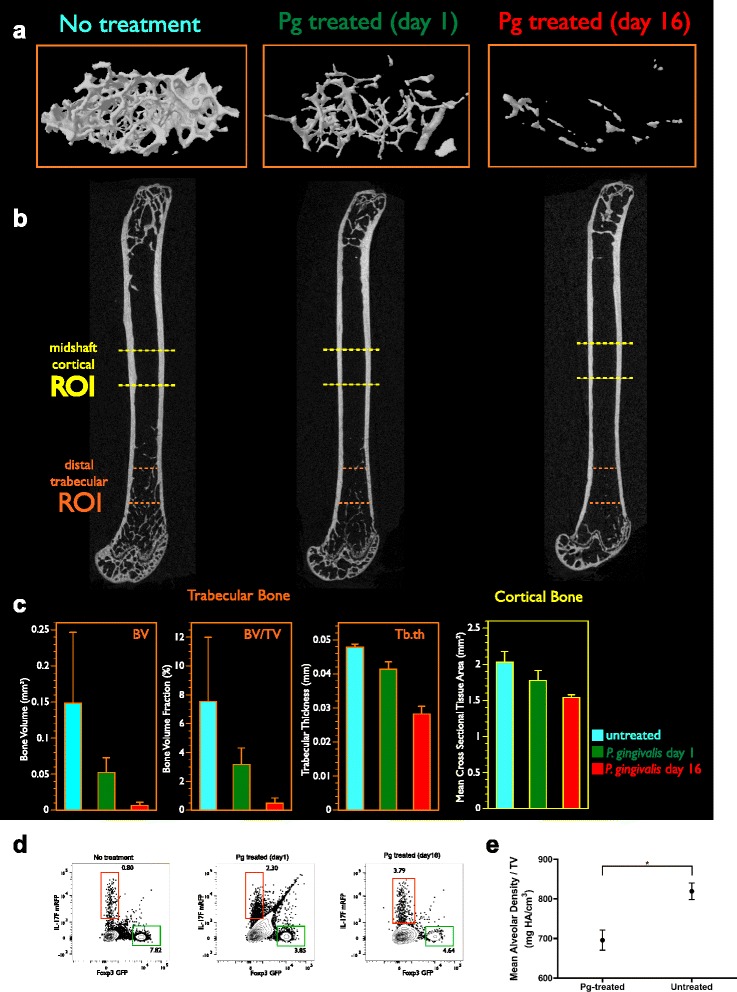
Bone loss in response to oral challenge with live *P. gingivalis.* Healthy suspensions of live *P. gingivalis* were mixed with CMC and brushed onto the gingival tissues of dual-reporter B6.DR1 mice. Legs were subjected to μCT analysis. **a** Proximal distal view of trabecular bone from mice sacrificed 1 or 16 days following treatment with *P. gingivalis* relative to those from untreated mice. **b** Orthogonal gray-scale images of femurs (not to scale). **c** Measurements of bone volume, bone volume fraction (bone volume/tissue volume), and trabecular thickness (*BV*, *BV/TV*, and *Tb.th* respectively) for trabecular bone and mean cross-sectional tissue area for cortical bone. Regions of interest used to calculate these values are indicated in **b**. **d** Flow cytometric analysis of cervical lymph nodes from corresponding mice at the time of sacrifice. Results expressed as mean percentage of CD3^+^CD4^+^ T cells expressing the IL-17F^mrfp^ (*red*) and Foxp3^gfp^ (*green*) reporter constructs. **e** Mean alveolar bone density in mice treated with *P. gingivalis* or left untreated (*N* = 3/group, *p < 0.05). *Error bars* in **c** = SEM; error bars in **e** = SD. *GFP* green fluorescent protein, *Pg Porphyromonas gingivalis*, *ROI* region of interest (Color figure online)

We used μCT analysis to measure bone density in the femurs of mice with or without *P. gingivalis* treatment. Femurs from naïve mice were compared with femurs from mice that were sacrificed 1 day or 16 days following seven daily brushings with *P. gingivalis*. Decreases in bone volume, bone volume fraction, and trabecular thickness (BV, BV/TV, and Tb.th respectively; Figs. 4a, c) indicating a loss of trabecular bone in the distal femur were detectable at 1 day following *P. gingivalis* treatment, and again at day 16 (green bars, Fig. 4c). Our calculations revealed a mean distal trabecular bone volume of 0.149 ± 0.098 mm^–3^ for femurs from untreated mice, 0.052 ± 0.020 mm^–3^ for those from mice 1 day following the last brushing with *P. gingivalis*, and 0.007 ± 0.005 mm^–3^ for those from mice at day 16. Bone volume fractions followed an almost identical trend and trabecular thicknesses were 0.048 ± 0.001, 0.041 ± 0.002, and 0.028 ± 0.002 mm respectively (Figs. 4a, c).

In addition to a loss of trabecular bone, we also measured a loss of cortical bone by selecting a mid-shaft region of interest (Figs. 4b, c) and measuring the mean cross-sectional tissue area. We found that the mean cross-sectional tissue area was 2.0 ± 0.15 mm^2^ for femurs from untreated mice, 1.8 ± 0.14 mm^2^ for femurs from mice 1 day following the last brushing, and 1.5 ± 0.04 mm^2^ for femurs from mice at 16 days after the last treatment.

We also analyzed the phenotype of the T cells in the cervical lymph nodes draining the oral cavity of each mouse as it was sacrificed for μCT analysis of its bone structure (Fig. 4d). We found that we could detect distinct increases in the percentage of CD4^+^ T cells expressing the Th17 phenotype of at day 1 and day 16 after treatment with *P. gingivalis* with percentages rising from less than 1 % to nearly 4 % by day 16.

Finally, as might be expected from what other laboratories have found in PD models, we also report that established periodontal disease and periodontal destruction was confirmed by detection of alveolar bone loss. As shown by μCT densitometric analyses, we detected bone loss between the first and second maxillary molars from *P. gingivalis*-treated compared with untreated mice. The mean alveolar bone density of *P. gingivalis*-treated mice was 695.8 ± 44.15 mg HA/cm^3^ vs untreated 819.4 ± 36.17 mg HA/cm^3^ (*N* = 3/group, *p* < 0.05) (Fig. 4e).

### Exacerbation of autoimmune arthritis following treatment with *P. gingivalis*

**Fig. 5:**
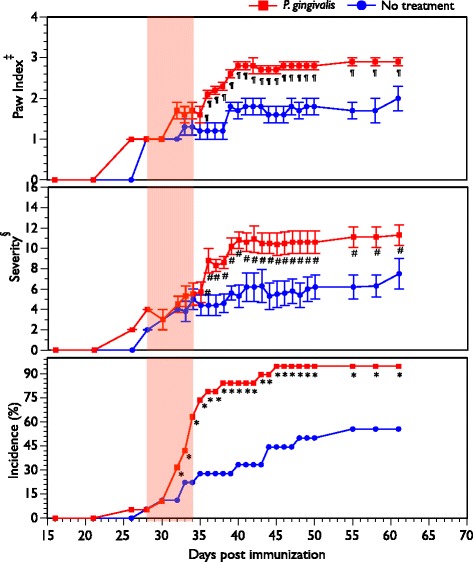
Exacerbation of autoimmune arthritis through brushing with *P. gingivalis*. Nineteen B6.DR1 mice were brushed daily for one week with *P. gingivalis* (*shaded pink area*) and 18 mice were left untreated. Clinical measures of paw swelling were obtained in all groups according to our standard protocol in which each paw is graded on a scale of 0–4 with 0 = no evidence of inflammation and 4 = maximally inflamed. *Statistically significant differences in arthritis incidence between the no-treatment and *P. gingivalis* groups (*p* < 0.04 by chi-squared test). ^¶,#^ *p* < 0.01 by Student’s *t* test. ^‡^Paw index is calculated as average number of inflamed paws per arthritic mouse. ^§^Severity index calculated as average severity per arthritic mouse. Results expressed as average ± SEM (Color figure online)

The B6.DR1 mouse was developed to study autoimmune arthritis on a common (B6) mouse background. We used our well-developed arthritis protocols to determine whether treatments with *P. gingivalis* would alter the expression of disease through measurements of clinical incidence, severity, paw index (the number of arthritic paws per arthritic mouse), and bone loss. In this experiment, the first two of the 39 CII/CFA-challenged mice had developed arthritis with a similar intensity at day 26 post immunization, so they were divided pairwise into the two treatment groups. Figure 5 demonstrates that brushing with *P. gingivalis* results in a dramatic acceleration of the disease expression, an enhancement of severity, and an increase in the paw index. There was a statistically significant increase in the percentage of arthritic mice in the *P. gingivalis* brushed mice even by the fourth of seven treatments. Differences in incidence were statistically significant almost immediately after the initiation of brushing and were maximal by day 34, which was the last day of brushing. Differences in severity and paw index were most evident a few days after the last brushing. By day 35, only 28 % of the untreated group were arthritic whereas 74 % of those brushed with *P. gingivalis* were arthritic.

### *P. gingivalis* treatment breaks resistance to autoimmune arthritis

**Fig. 6:**
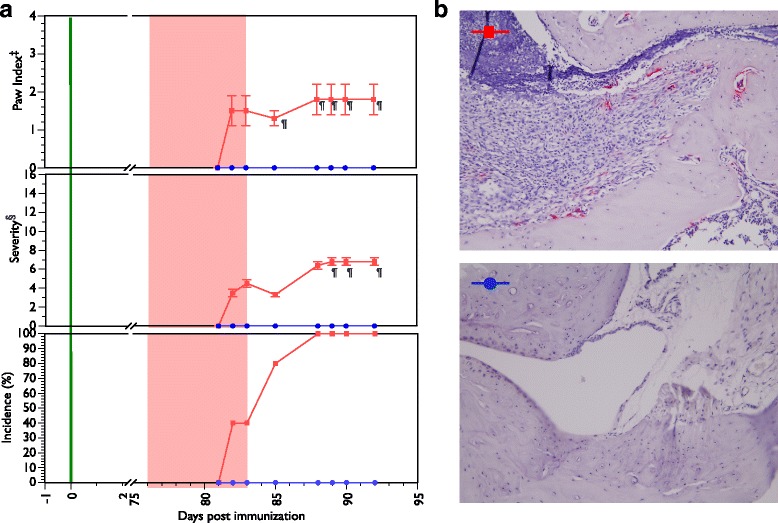
Breaking resistance to autoimmune arthritis by brushing with *P. gingivalis.* **a** Nine B6.DR1 mice which had not developed arthritis at 76 days post CIA challenge (*green vertical line*) were used for this experiment. Five mice (*red squares*) were brushed daily for 7 days (*shaded pink area*) with *P. gingivalis* and four mice (*blue circles*) were left untreated. ^‡^Paw index is calculated as average number of inflamed paws per arthritic mouse. ^§^Severity index calculated as average severity per arthritic mouse. **b** TRAP staining of knee joints from *P. gingivalis*-treated and untreated mice. Magenta staining identifies TRAP^+^ osteoclasts. ^¶^ *p* < 0.05 by Student’s *t* test. Results expressed as average ± SEM (Color figure online)

We challenged a large group of mice with bovine CII emulsified in CFA (CII/CFA) in order to elicit inflammatory autoimmune arthritis. As in many previous experiments, a small percentage (5–10 %) of the mice exhibited a resistance to CIA and did not show any clinical signs of disease. Seventy-six days after CII/CFA challenge we brushed five of nine CIA-resistant mice with 10^6^ CFU of live *P. gingivalis* cells daily for 7 days. By 5 days after the last treatment, all five mice had developed arthritis whereas all four of the untreated mice remained CIA resistant (Fig. 6a). This disease was characterized by typical inflammatory infiltrates including the presence of numerous TRAP^+^ osteoclasts in the tissues of *P. gingivalis*-treated mice but a complete absence of pathology in the untreated animals (Fig. 6b). While the severity of disease was not as great as in the previous experiment (Fig. 5, middle panel), the disease expression kinetics was similar with two of the five mice developing signs of arthritis before the last treatment with *P. gingivalis*.

### Bone loss in *P. gingivalis*-treated arthritic mice

**Fig. 7:**
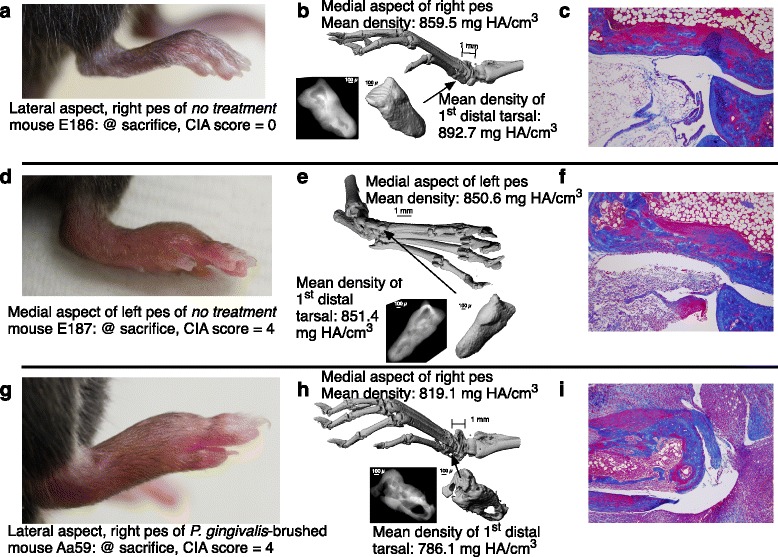
Bone loss in pedes of *P. gingivalis*-brushed arthritic mice. μCT analysis of one non-involved (score = 0) pes from a no-treatment mouse, one maximally inflamed pes (score = 4) from a no-treatment mouse, and one maximally inflamed (score = 4) pes from a mouse brushed with *P. gingivalis*. **a**, **d** & **g** Photographs of pedes taken at euthanasia. **b**, **e** & **h** 3D reconstructions of bone morphology of each pes and of the first distal tarsal with bone densities calculated according to an algorithm written specifically for that purpose. **c**, **f** & ﻿**i** Masson’s trichrome stained sections of each corresponding ankle joint

We used μCT analysis to examine the structure of bones in the limbs of arthritic mice with or without *P. gingivalis* treatment. We used specific bone-density measurements to quantify the bone loss that happens as a result of the disease process in CIA. When analyzing the density of the entire paw, we were cautious in using specific anatomical landmarks to be sure that we were measuring identical structures on each sample analyzed, and compared paws that had both identical clinical scores and identical onset dates so that the disease duration over which the measured paw had experienced inflammation was comparable. Our analyses of pedes (Fig. 7) revealed that, despite apparent clinically similar severities (Fig. 7d & g), paws from mice that had been brushed with *P. gingivalis* (Fig. 7h) appeared to have greater damage and a lower overall bone density than those that were not treated (﻿Fig. 7e). An uninvolved pes from a mouse in the no-treatment group (7b) had an average bone density of 859.5 mg HA/cm^3^, a fully arthritic (CIA score of 4) pes from another no-treatment mouse (Fig. 7e) had an average bone density of 850.56 mg HA/cm^3^, whereas a fully arthritic pes from a *P. gingivalis*-treated mouse (Fig. 7h) had an average bone density of 819.1 mg HA/cm^3^.

**Fig. 8:**
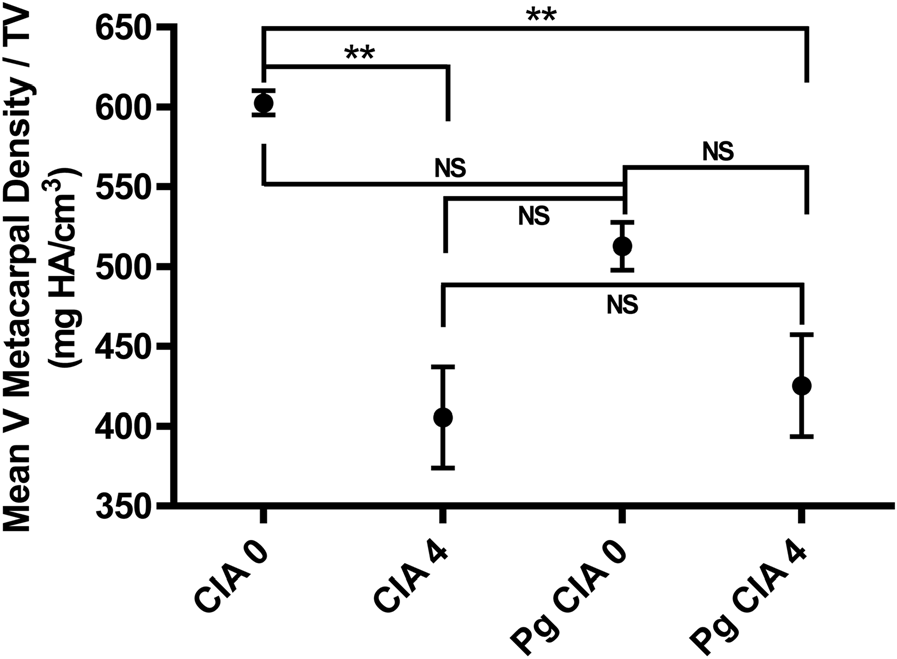
Bone loss in manus of *P. gingivalis*-brushed arthritic mice. μCT analysis of three metacarpal bones each from digit V of noninvolved (score = 0) manus from mice with/without treatment with *P. gingivalis*, and three metacarpal bones each from digit V of maximally inflamed (score = 4) manus from mice with/without treatment with *P. gingivalis. Error bars* = SD. **p < 0.01. *NS* not statistically significant. *Pg Porphyromonas gingivalis*

In addition to evaluating the overall density of the entire paw structure, we determined that it was helpful to focus the measurement on specific bones within the paw to give us a more sensitive measure of bone loss. As an example, for the pedes we measured bone mineral density analysis of the first distal tarsal bone. The first distal tarsal bone is located in the middle of the pedes where the maximum pathology (clinical arthritic score) is observed in the CIA model. This bone (inset, Fig. 7b, e & h) has a high ratio of joint surface to total bone volume, and is easily identified. The mean bone mineral density of the first distal tarsal appears reduced in the *P. gingivalis*-treated mouse with CIA clinical score 4 (inset, Fig, h: bone density 786.1 mg HA/cm^3^), relative to that of both the uninvolved pes (Fig. b: bone density 892.7 mg HA/cm^3^) and arthritic pes (Fig. e: bone density 851.4 mg HA/cm^3^). Both 3D reconstruction and radiographic imaging of the first distal tarsal bone suggested significant structural damage in the pes of the arthritic *P. gingivalis*-treated mouse compared with the control mice without *P. gingivalis* treatment. Representative histology of ankle sections (Fig. 7c, f & i) underscores the inflammatory cellular infiltrates observed in the arthritic mice. However, there were insufficient numbers of comparable pedes in each group to provide a statistical analysis, so we selected the metacarpal bone of the fifth digit of the manus of a larger number of mice (four groups of three each) in order to apply statistical power to the study. The effect of *P. gingivalis* treatment in relation to the development of autoimmune arthritis (maximally inflamed manus, score = 4 vs no involvement, score = 0) on the mean bone density of the V metacarpal is shown in Fig. 8. While it did not reach statistical significance, treatment with *P. gingivalis* triggered a trend towards bone loss in the absence of clinically detectable autoimmune arthritis. Moreover, development of autoimmune arthritis was characterized by significant loss in the bone density of the V metacarpal, as shown by μCT densitometric analysis (CIA score 0, bone density 602.7 ± 13.29 mg HA/cm^3^; CIA score 4 bone density 405.5 ± 44.71; *P. gingivalis* CIA score 0, bone density 512.8 ± 25.83; and *P. gingivalis* CIA score 4, bone density 425.5 ± 55.1; *N* = 3 manus/group; *p* < 0.01).

## Discussion

Our study was designed to further understanding of the underlying mechanisms that link PD to RA. Our goal was to do this work in the context of a standardized common mouse strain, C57BL/6, on which the vast majority of transgenes, knockins, and knockouts are found. However, where arthritis is concerned, some laboratories have reported variability in the CIA susceptibility of C57BL/6 mice [21, 22], which may represent manifestations of differing intestinal flora [23]. We have circumvented this problem by generating an I-A°/I-E° mouse on the C57BL/6 background that expresses a chimeric mouse/human RA susceptibility allele HLA-DR1(*0101) as a transgene. Recent research has suggested that this same DR1 restriction element may also serve as a susceptibility allele for periodontal disease [24, 25], providing a unique opportunity for use as an animal model that is perfectly suited for the analysis of linkages between the two disease processes. While there has been one study in which HLA-DR4 tg mice were used to look at the role of citrullinated human α-enolase [11], to our knowledge no HLA-DR1 mice have been challenged with *P. gingivalis* to assess its role in ACPA development or modulation of arthritis.

There is abundant anecdotal evidence surrounding the development of arthritis and other diseases following dental procedures [26]. The prevailing hypothesis is that chewing, brushing, and even the simplest dental procedures can cause transient bacteremia and disseminate pathogens in the bloodstream that may consecutively trigger systemic health challenge, especially in susceptible hosts (i.e. immunocompromised patients). Our brushing technique and model system was designed to mimic the introduction of oral flora, including pathogens, into the periodontal tissues of mice bearing the human class II MHC susceptibility alleles, with stiff bristles attached to a syringe through which healthy cultures of the Gram-negative anaerobe *P. gingivalis* is delivered in a slurry of CMC. We wish to establish a chronic *P. gingivalis* periodontal infection that, compared with the ligature [27] and gavage [12] models, does a better job of recreating the systemic pulses of these oral pathogens into the mouse. The ligature-induced PD model involves mechanical trauma caused by ligature insertion and results in alveolar bone loss, most likely due to the trauma (and possibly the nonspecific bacterial plaque accumulation) that differs from the classic bacterial etiology and pathogenesis of PD independent of mechanical trauma. Furthermore, the gavage of *P. gingivalis* may achieve an effective systemic but transient bacterial challenge, while brushing of *P. gingivalis* may facilitate the establishment of a chronic infection of *P. gingivalis* in the periodontal tissues and our findings suggest that it can trigger significant pathological processes and autoimmune response distinct and spatially removed from the inflammation and alveolar bone damage seen in the ligature model.

Both PD and RA are known to be driven by the development of proinflammatory Th17 cells which can produce the cytokines known to be associated with the destructive environment found in the RA joint and in periodontal tissues [2, 28]. Cytokines produced by Th17 cells can drive the differentiation of osteoclasts from precursors and these osteoclasts can tip the balance in the direction of bone resorption [29]. In addition to osteoclast increase, a recent study has shown that IL-17A can decrease osteoblast formation resulting in a net bone loss [30].

Our data demonstrate that a burst of Th17 cell generation takes place systemically because we can see a rise in the percentage of CD4^+^ T cells expressing the Th17 phenotype and a subsequent rise in systemic levels of IL-17A. We propose that this shift in T-cell phenotype may give rise to both an increase in osteoclast formation and a decrease in osteoblast formation, each of which may contribute to the bone loss that we measured in our μCT analyses.

Our analysis of serum cytokines demonstrates that TNF-α, IL-1β, and IL-17A are all produced in response to treatment with *P. gingivalis*, whereas other cytokines such as IL-10 and IFN-γ are not produced, making any changes in Th1-mediated activities unlikely. We expected to see an increase in IL-6, a cytokine which works in concert with TGF-β to activate the transcription factor ROR-γt and drive the differentiation of Th0 cells into the Th17 program. It is possible that the IL-6 may have been expressed locally at the site of infection, or that it was an early event which occurs prior to day 7.

If indeed a rise in Th17 levels is necessary to bring about autoimmunity in the face of subclinical CIA, it would be interesting to determine whether treatment with a Th17-inducing prokaryote such as *P. gingivalis* might have a similar effect on a Th1-dependent disease such as antigen-induced arthritis.

One of the more interesting findings in our study was that brushing with *P. gingivalis* not only increased arthritis incidence and hastened its onset, but the paw index and disease severity were also enhanced by statistically significant values. At the end of the study, untreated mice had an average of exactly two involved paws per mouse whereas those brushed with *P. gingivalis* had an average of 3.1 arthritic paws. The mechanisms driving the paw index are as yet unknown, but one may presume that increasing the paw index might be similar to the thresholds that drive incidence in a given study; once that threshold is crossed, an individual mouse or individual paw may become arthritic.

One of the more intriguing findings in our study was the evidence suggesting a role of HLA-DR1 in the generation of ACPAs. There is no shortage of clinical studies [31–34] which have suggested that not only does ACPA generation correlate well with the presence of the SE, but that the fine specificity of ACPA epitopes may also be shaped by it [35]. We asked whether the presence of a transgenic expression of the SE could provide the necessary restriction element to allow a mouse to produce ACPAs in response to brushing with *P. gingivalis*. We chose to use a clinical diagnostic kit that is approved for use in humans in order to determine whether the chimeric mouse/human restriction element could drive the generation of ACPAs in this model. Our results clearly demonstrated that ACPAs were generated in response to treatment with *P. gingivalis*, but only in mice bearing the DR1 transgene and not in WT (B6) mice. This suggests that the ACPAs being measured are linked to the expression of the HLA-DR1 restriction element. Furthermore, we also demonstrated that the generation of ACPAs in this model required the expression of prokaryotic PAD enzyme presumably to drive the citrullination of host proteins. Together, these findings demonstrate a need for both prokaryotic PAD and the HLA-DR1 restriction element in order to drive the robust generation of ACPAs in this model.

The end result of both periodontal disease and RA is loss of structural integrity and function. In the case of periodontal disease, this results in tooth loss; and in the RA joint, cartilage is destroyed and bones are eroded resulting in an ankylosis of the joint and loss of function. While RA-induced bone loss and periodontal tooth loss are very well documented in the clinical literature, and bone loss has also been reported in animal models of experimental arthritis [36], to our knowledge direct measures of nonalveolar bone loss resulting from the introduction of an oral pathogen alone have not been documented. We found a dramatic loss of trabecular bone and even a modest loss of cortical bone following treatment with *P. gingivalis*. In addition, μCT analysis in a limited number of mice revealed bone loss dramatic enough to result in actual fenestrations in the proximal tibia–fibula. While this finding was limited to a small number of mice, it highlighted the dramatic bone losses that could result from treatment with *P. gingivalis*. We would propose that the repeated systemic pulses of *P. gingivalis* in this model drives a sufficient systemic Th17 response capable of reducing osteoblast formation and generating sufficient numbers of osteoclasts to reduce bone volume in the extremities.

One of the most interesting findings in this study was that mice which were otherwise resistant to the development of collagen-induced arthritis could be induced to rapidly develop clinical signs of autoimmune arthritis following oral exposure to *P. gingivalis*. Typical CIA induction experiments result in between 80 and 100 % incidence among positive control mice. While it is unknown why some of the mice treated exactly the same as others do not develop disease, we were very interested to see that mice which had not develop disease for more than 10 weeks after challenge with type II collagen emulsified in CFA could be rapidly induced to do so following oral exposure to *P. gingivalis*. This suggests that if all of the disease-inducing factors are already present, then an oral infection could possibly tip the balance in favor of clinical expression of disease. The rapid onset of disease expression (within 2 days) suggests that the generation of ACPAs or antibodies directed at *P. gingivalis* would not likely play a role in this induction. It is more likely that this response is driven by innate immune pathways, and it will be of great interest to elucidate the mechanism(s) driving this interesting finding.

## Conclusions

The B6.DR1 mouse allows us to examine the influence of periodontal pathogens in the context of a relevant human MHC class II restriction element HLA-DRβ1 during the development of autoimmune arthritis. This restriction element provides the necessary framework for the generation of ACPAs in response to infection with an oral pathogen. From this study, it is clear that treatment with the periodontal disease pathogen *P. gingivalis* not only exacerbates ongoing autoimmunity, but may also provide a trigger for disease development if other disease-promoting conditions are present.

## Abbreviations

μCT: Microcomputed tomography
ACPA: Anti-citrullinated protein antibody
CFA: Complete Freund’s Adjuvant
CFU: Colony-forming unit
CII: Type II collagen
CMC: Carboxymethyl cellulose
dPAD: PAD-null mutant strain of *P. gingivalis*
ELISA: Enzyme-linked immunosorbent assay
GFP: Green fluorescent protein
HLA: Human leukocyte antigen
mRFP: Mono-red fluorescent protein
PAD: Peptidyl arginine deiminase
PBS: Phosphate-buffered saline
PD: Periodontitis
pPAD: PAD derived from *P. gingivalis*
RA: Rheumatoid arthritis
SE: Shared epitope
SEM: Standard error of the mean
VOI: Volume of interest
WT: Wild type
